# Hydrological regime and niche partitioning drive fungal community structure and function in arid wetlands sediments of South Africa

**DOI:** 10.1007/s11356-025-36592-0

**Published:** 2025-06-06

**Authors:** Henry Joseph Oduor Ogola, Grace Nkechinyere Ijoma, Joshua Nosa Edokpayi

**Affiliations:** 1 Department of Environmental Sciences (DES), College of Agriculture and Environmental Sciences (CAES), University of South Africa (UNISA), Florida Campus, Roodepoort, 1709 South Africa; 2 Water and Environmental Management Research Group, Faculty of Science, Engineering and Agriculture, University of Venda, Thohoyandou, 0950 South Africa

**Keywords:** Fungal communities, Shotgun metagenomics, Hydrological variability, *Mucoromycota*, *Ascomycota*

## Abstract

Arid wetlands are ecologically significant yet understudied ecosystems shaped by extreme conditions and hydrological variability. However, the structure and ecological functional of fungal communities in these habitats remain poorly understood, especially in southern Africa. This study integrated shotgun metagenomics, FUNGuild functional profiling, and multivariate analyses to examine fungal diversity, functional composition, and environmental drivers in seasonal and permanent arid wetlands of South Africa. Distinct fungal assemblages emerged, primarily shaped by hydrological regimes and ionic stress. Seasonal wetlands were dominated by *Mucoromycota* (79%), particularly arbuscular mycorrhizal (AM) fungus (*Rhizophagus,* 62%), while permanent wetlands had higher *Ascomycota* (54%), with *Aspergillus* (50%) prevalent in oxygen-limited sediments. Although alpha diversity showed no significant difference, beta diversity confirmed significant mycobiome differentiation. Total dissolved solids (TDS), electrical conductivity (EC), and salinity were key predictors of fungal composition, with TDS the strongest determinant (*p* < 0.01). Functional guild analysis highlighted niche differentiation, with saprotrophs dominating permanent wetlands (59.7% vs. 21.5%; *p* < 0.05), while symbiotrophs, particularly AM fungi, were enriched in seasonal wetlands (69.3% vs. 36.1%; *p* < 0.001). Indicator taxa identified via LefSe (LDA > 3, *p* < 0.05) and random forest modeling included *Rhizophagus, Trichoderma, Fusarium,* and *Entomophthora* in seasonal wetlands, and *Aspergillus* in permanent wetlands. This study provides the first integrative insight into fungal ecology in South Africa’s arid wetlands, demonstrating that hydrological regime shapes fungal structure and function through environmental filtering and niche specialization, with implications for guiding conservation and adaptive management of these fragile ecosystems.

**Supplementary Information:**

The online version contains supplementary material available at 10.1007/s11356-025-36592-0.

## Introduction

Arid wetlands, shaped by episodic rainfall and high evaporation rates, constitute extreme ecosystems defined by highly variable hydrological and physicochemical regimes (Grenfell et al. 2022). In such systems, the interplay of intermittent drought and inundation (i.e., water pulses), salinity fluctuations, and nutrient limitations (oligotrophy) imposes strong selective pressures on both macro- and microbial communities, influencing their structure and functional potential (Austin et al. 2004; Dube et al. 2023). Although these wetlands occur across diverse global regions, including southern Africa, Australia, southern Europe and parts of Asia (Williams 2000; Grenfell et al. 2022; Jiménez-Melero et al. 2023; Wu et al. 2024), ecological research has largely focused on vegetation and hydrology. In contrast, the soil microbiome, particularly fungal communities, has received comparatively little attention, despite increasing recognition of its role in ecosystem functioning, including organic matter turnover, nutrient cycling, and resilience to environmental stressors.

Fungi play essential roles in wetland ecosystems as decomposers, symbionts, and key agents of nutrient cycling. In arid wetlands, microbial community assembly is frequently governed by niche partitioning, wherein taxa occupy discrete ecological roles that minimize competition and enhance resource utilization (Tidimalo et al. 2024). This partitioning is primarily driven by environmental gradients, including moisture availability, ionic stress, and organic matter content, which collectively shape both fungal taxonomic composition and functional guild distributions (Wu et al. 2013; Chen et al. 2024). Under desiccation-prone conditions, xerotolerant fungi exhibit metabolic plasticity, enabling survival during extended dry periods. Concurrently, symbiotrophic taxa such as arbuscular mycorrhizal fungi (AMF) enhance plant nutrient acquisition under fluctuating moisture regimes (Chen et al. 2024). In contrast, saprotrophic fungi typically dominate in more hydrologically stable wetlands, where they facilitate organic matter decomposition under anaerobic or oxygen-limited conditions (Onufrak et al. 2020). These observations underscore the need for detailed investigations into fungal niche specialization in response to hydrological and geochemical constraints in arid wetland soils.

Building upon the understanding of niche specialization in response to hydrological and physicochemical factors, recent studies further demonstrate that fungal communities in arid and semi-arid ecosystems exhibit strong habitat differentiation along environmental gradients. For example, Tidimalo et al. (2024) reported that fungal diversity in semi-arid soils of Botswana was driven by soil pH, organic carbon, and ion concentrations, with taxa such as *Pleosporales* (*Teichospora)*, *Hypocreales* (*Bisifusarium)*, *Sordariales* and *Filobasidiales* (*Naganishia*) exhibiting distinct ecological preferences. Similarly, Chen et al (2023) found that seasonal shifts in wetland hydrology influenced fungal composition, with chytrid fungi (*Rhizophydiaceae*, *Rhizophydiales*) thriving in dry periods and *Eurotiales* and *Saccharomycetales* dominating during inundation. Factors such as temperature and ammonium (NH_4_^−^) concentrations influenced fungal communities in the dry season, while in the wet season, factors such as total nitrogen (TN), conductivity (EC), TOC, phosphorus (TP), pH, and temperature were more significant (Chen et al. 2023). In China’s arid Songnen Plain, fungal richness declined following wetland degradation, with community shifts closely linked to reductions in TOC, TN, and available phosphorus (Yan et al. 2024). These findings highlight the importance of niche differentiation in shaping fungal community structure under hydrological stress.

Despite the recognized ecological importance of fungi, microbial investigations in South Africa’s arid wetlands, primarily situated in the Namaqualand, Karoo, and Limpopo regions, remain limited. Existing research has predominantly concentrated on macroorganisms and abiotic system attributes, such as hydrological variability and geomorphological features (Sieben et al. 2017; Grenfell et al. 2022; Dube et al. 2023; Ogola et al. 2024), leaving a significant gap in our understanding of fungal contributions to ecosystem resilience and biogeochemical functioning. To address this gap, we employed shotgun metagenomics, a culture-independent approach, to characterize fungal diversity, functional guild composition, and niche partitioning across seasonal and permanent arid wetlands in South Africa. Specifically, we aimed to: 1) characterize the mycobiome composition within these wetland types; 2) assess functional guild distributions and their ecological roles using FUNGuild software (Nguyen et al. 2016); and 3) evaluate the influence of hydrological regimes and key environmental drivers, particularly total dissolved solids (TDS) and salinity, on fungal community structure. By elucidating how salinity and other hydrochemical stressors shape fungal specialization under fluctuating water regimes, this study advances our understanding of microbial adaptation and ecosystem resilience in extreme wetland environments.

## Materials and methods

### Site description

Sediment samples were collected from 10 unprotected, natural arid wetlands across the Capricorn, Mopani, Vhembe, Sekhukhune, and Waterberg districts in the arid northeastern region of South Africa's Limpopo Province. This region experiences a summer rainfall climate, characterized by hot, humid conditions from October to April and cooler, drier winters. During summer, temperatures often exceed 40 °C, with rainfall reaching up to 1500 mm. In contrast, winter temperatures average around 20 °C, with rainfall typically around 200 mm (Matimolane et al. 2024). Sampling was conducted during the dry winter season (September–October 2020), targeting ten wetlands, six of which are classified as permanent (retaining water year-round), while the remaining four are seasonally ephemeral.

Detailed descriptions of the sampling sites and environmental parameters (including geographic locations and hydrological features), sediment sampling protocols (including site selection, sample collection strategy, and composite preparation), and physicochemical profiling have been described comprehensively by Ogola et al. (2024).

### Sampling and physicochemical profiling

In brief, from each wetland, surface sediment samples (0–10 cm depth) were collected using a Kajak corer dredge (KC-Denmark). At each site, five subsamples were randomly collected within a 2 m × 2 m area and homogenized to form one composite sample. This process was repeated at three spatially separated zones per wetland, resulting in a total of 30 composite sediment samples (3 replicates per wetland × 10 wetlands). Immediately after collection, sediment samples were transferred into sterile containers, stored on ice (~ 4 °C), and transported to the laboratory. Upon arrival, approximately 20 g of each composite sample was placed in sterile centrifuge tubes, flash-frozen in liquid nitrogen, and stored at − 80 °C until DNA extraction.

During sampling, field-based in situ measurements were taken using a Hanna HI9828 multi-parameter ion-specific meter (Hanna Instruments, South Africa) to record water temperature, pH, salinity, total dissolved solutes (TDS), dissolved oxygen (DO), and electrical conductivity (EC). Biological oxygen demand (BOD₅) was also assessed using the APHA 5210B standard method. Sediment geochemical properties were determined from a 1:5 soil-to-deionized water slurry, shaken for 2 h and left to settle for 12 h. The supernatant was analyzed for pH (Adwa AD11 pH meter), salinity, EC, and TDS. A complete methodological account of the geochemical analyses is available in Ogola et al. (2024). The summary of the sampling sites and physicochemical analysis of water and sediment samples is provided in Supplementary Material Table S1.

### DNA extraction, libraries preparation and shotgun sequencing

To minimize experimental biases and ensure data reproducibility, total environmental DNA was extracted in triplicate from each composite sediment sample using the DNeasy® PowerSoil Pro Kit (Qiagen N.V., Hilden, Germany), following the manufacturer’s protocol. The resulting DNA replicates from each wetland were pooled prior to downstream applications. DNA quality and concentration were evaluated using a NanoDrop 2000 spectrophotometer (Thermofisher Scientific, Waltham, MA, USA) and a Qubit™ dsDNA BR Assay Kit (Thermo Fisher Scientific, Waltham, MA, USA) respectively. Only samples with A_260_/A_280_ ratios between 1.8 and 2.0 and DNA concentrations ranging from 20 to 150 ng/μl were selected for library preparation, thresholds aligning with the Illumina NGS Best Practices and Recommendations (Bronner and Quail 2019).

DNA libraries were prepared using the Nextera XT® DNA Library Preparation Kit and IDT Unique Dual Indexes® (Illumina, San Diego, CA, USA) with 1 ng of input DNA. The libraries were barcoded, amplified, and purified using AMpure XP® beads (Beckman Coulter, Brea, CA, USA). Library quality was evaluated using the Qubit™ dsDNA HS Assay Kit. Sequencing was performed on an Illumina NextSeq 2000® platform (2 × 150 bp) at CosmosID Inc. (Germantown, MD, USA), yielding between 23,618,694 and 42,062,226 paired-end reads per sample. Raw sequencing data are available in the NCBI SRA database under BioProject ID PRJNA972844, with SRA accession numbers SRX20358958–SRX20358949.

### Bioinformatic analyses

#### Quality filtering, trimming and assembly

Raw sequencing reads were imported into KBase platform (https://www.kbase.us/) for downstream curation (quality filtering, trimming and assembly) using its suite of bioinformatics tools (Arkin et al. 2018)**.** Initial quality assessment was conducted using FastQC v0.12.8, providing detailed insights into overall read quality, average read length, duplicate content, and GC content distribution in each sample. To enhance sequence quality, NexteraPE adapter sequences were removed, and low-quality reads were trimmed using Trimmomatic v0.39 (Bolger et al. 2014)**.** Parameters included a minimum Phred score of 30, a minimum read length of 36, and a sliding window approach with a window size of 4 and quality cutoffs of 15. The quality-filtered reads were assembled using Megahit v1.1.2 (Li et al. 2016), with default parameters on the KBase platform. Contigs with a length of at least 300 bp were selected as the final assembly outcome, after which the contigs were subjected to taxonomic classification, prediction and gene annotation.

#### Kaiju taxonomic classification

Taxonomic classification of fungal communities to species level of the assembled contigs was performed using the KBase Classify Taxonomy of Metagenomic Reads with Kaiju App v1.9.0 (Menzel et al. 2016)**.** The analysis employed the greedy-5 mode against the curated NCBI BLAST nr + euk reference database, encompassing protein sequences from Bacteria, Archaea, Viruses, Fungi, and microbial eukaryotes (Arkin et al. 2018)**.** Quality-filtered sequences were assigned taxonomic IDs and annotated with functional information based on reference mappings. Operational taxonomic units (OTUs) were identified and categorized according to their relative abundance and taxonomic assignments. These taxonomic classifications were subsequently used for downstream analyses, including fungal community diversity profiling and visualization.

### Statistical analyses

All statistical analyses were performed using R (v4.4.1) (R Core Team 2021), with core analyses conducted through the *microeco* package (v1.13–0) (Liu et al. 2021). Taxa-level relative abundances, including core fungal community composition at genus-level analysis were visualized via stacked barplots, heatmaps and Venn diagrams using ggplot2 package (Wickham 2016). To assess mycobiome diversity within and among arid wetland sites, alpha diversity was quantified using Shannon and Abundance-based Coverage Estimator (ACE) richness based on taxonomically classified contigs derived from Kaiju outputs. To control for sequencing depth variability, all relative abundance data were rarefied to the lowest read count observed across samples before conducting diversity and downstream analyses.

Differences in fungal diversity and taxonomic composition between wetland types were assessed using the Wilcoxon rank-sum test, followed by Tukey’s Honest Significant Difference (TukeyHSD) method for post hoc comparisons. Beta diversity was analyzed by calculating Bray–Curtis dissimilarity distances using *vegdist* function from the *vegan* package (v2.6–10) in R (Oksanen et al. 2019), community-level differences visualized through Principal Coordinate Analysis (PCoA). Statistical testing for compositional dissimilarities among wetland types was carried out using both Permutational Multivariate Analysis of Variance (PERMANOVA) and Analysis of Similarities (ANOSIM), implemented through the *adonis* and *anosim* functions in vegan, based on 999 permutations. To ensure that observed differences in beta diversity were not confounded by group dispersion, we performed Permutation Analysis of Multivariate Dispersion (PERMDISP) using the *betadisper* function from the same package.

Differentially abundant fungal OTUs (biomarkers) between wetland groups were identified using linear Discriminant Analysis (LDA) coupled with the effect size (LEfSe) (Segata et al. 2011). The *ldamarker()* function incorporating Kruskal–Wallis testing and default cross-validation settings, was used via the Galaxy-based LEfSe pipeline (https://usegalaxy.org), applying a threshold of LDA score > 2 and *p* < 0.05. In parallel, random forest (RF) models were used to assess genus-level taxonomic differentiation and classification accuracy using the *rfPermute* package (v2.5.4) (Archer 2025), with settings of 3000 permutations and 1000 trees. Taxa were ranked based on mean decrease in Gini impurity (meanDecreaseGINI), and the top ten genera were statistically evaluated using Kruskal–Wallis or Mann–Whitney U tests.

To investigate environmental influences on fungal community structure, distance-based redundancy analysis (dbRDA) was performed using Bray–Curtis distances of sediment-associated fungal communities. Spearman correlation coefficients (*ρ* > 0.6, *p* < 0.05) were computed using the *corrplot* package (v1.8.0) (Wei and Simko 2024) to examine associations between environmental variables and fungal beta diversity. Additionally, Mantel tests were conducted using the mantel function in vegan package to assess pairwise Spearman correlations between fungal composition and individual environmental factors. To identify key environmental drivers of fungal beta diversity, random forest regression models were implemented using the *rfPermute* function, with 5000 permutations and 2000 trees. To avoid multicollinearity, among highly correlated predictors (|r|≥ 0.70), only those with the highest percentage increase in mean squared error (%IncMSE) were retained. Final models were optimized via backward selection until no further gains in explained variance were observed. Predictor importance and significance were extracted from the final models, and linear regression analyses were used to validate associations between Bray–Curtis distances and key environmental variables.

Finally, the functional roles and trophic modes (i.e., pathotroph, symbiotroph, or saprotroph) of fungal communities were inferred by annotating OTUs using the FUNGuild database https://github.com/UMNFuN/FUNGuild, accessed 2 February 2025) (Nguyen et al. 2016). FUNGuild assigns fungal taxa to ecological guilds based on curated literature classifications, encompassing twelve functional categories thereby enabling a comprehensive ecological assessment of fungal community composition across wetland types.

## Results and discussion

### Metagenomic sequencing quality and taxonomic resolution

Raw read counts across samples exhibited substantial variation, ranging from 23.6 million to 42.1 million reads, with a mean of 32.8 ± 5.6 million reads (Table 1). Following stringent quality filtering, over 80% of reads were retained in most samples. Contig assembly metrics also differed markedly, with seasonal wetlands generally yielding more contigs than permanent ones, suggesting greater microbial complexity. N50 values, indicative of assembly continuity, ranged from 6,707 to 20,107 bp (mean = 13,244 ± 4,212 bp), with samples S8 and S2 exhibiting particularly high contiguity.

**Table 1:** Summary of global sequencing statistics, assembly and taxonomic classification

Sample	Raw reads	Quality reads	Megahit Assembly				Kaiju taxonomic classification		
			No. of Contigs	Total length (bp)	GC (%)	N50 (bp)	Bacterial reads (%)	Eukarya reads (%) ^a^	Viral reads (%)
Seasonal wetland									
S1	34,429,730	29,117,492	16,031	97,592,641	47.4	8,238	1,127,033 (97.8)	22,541 (1.93)	3,728 (0.32)
S3	41,954,238	31,254,266	11,174	69,346,611	42.8	8,373	1,061,338 (98.1)	16,613 (1.54)	4252 (0.39)
S4	33,272,268	32,559,506	8,173	44,449,322	43.6	6,707	1,097,969 (93.3)	78,948 (6.67)	959 (0.08)
S8	39,417,602	32,437,842	5,988	55,684,101	49.0	20,107	1,373,820 (97.4)	34,190 (2.42)	2,373 (0.17)
Permanent wetland									
S2	25,176,054	20,171,024	6,020	56,609,334	51.4	18,024	855,396 (98.0)	13,535 (1.55)	3552 (0.41)
S5	23,618,694	17,221,588	8,220	52,008,987	45.8	9,678	617,472 (97.1)	15,792 (2.48)	2,905 (0.46)
S6	27,991,602	27,152,208	8,683	49,325,157	46.0	7,030	1,092,919 (98.5)	13,858 (1.25)	2,676 (0.24)
S7	27,052,694	26,249,204	4,847	32,890,255	46.7	14,212	1,132,398 (98.8)	11,370 (0.99)	2,235 (0.20)
S9	42,062,226	35,833,994	7,452	46,008,202	54.0	8,493	1,486,112 (98.7)	19,074 (1.27)	1,158 (0.08)
S10	33,650,822	27,697,006	11,633	87,600,269	48.6	12,350	1,134,405 (93.7)	74,534 (6.16)	1,458 (0.12)

^a^ A total of 687 fungal OTUs were generated for downstream analysis

**Fig. 1:**
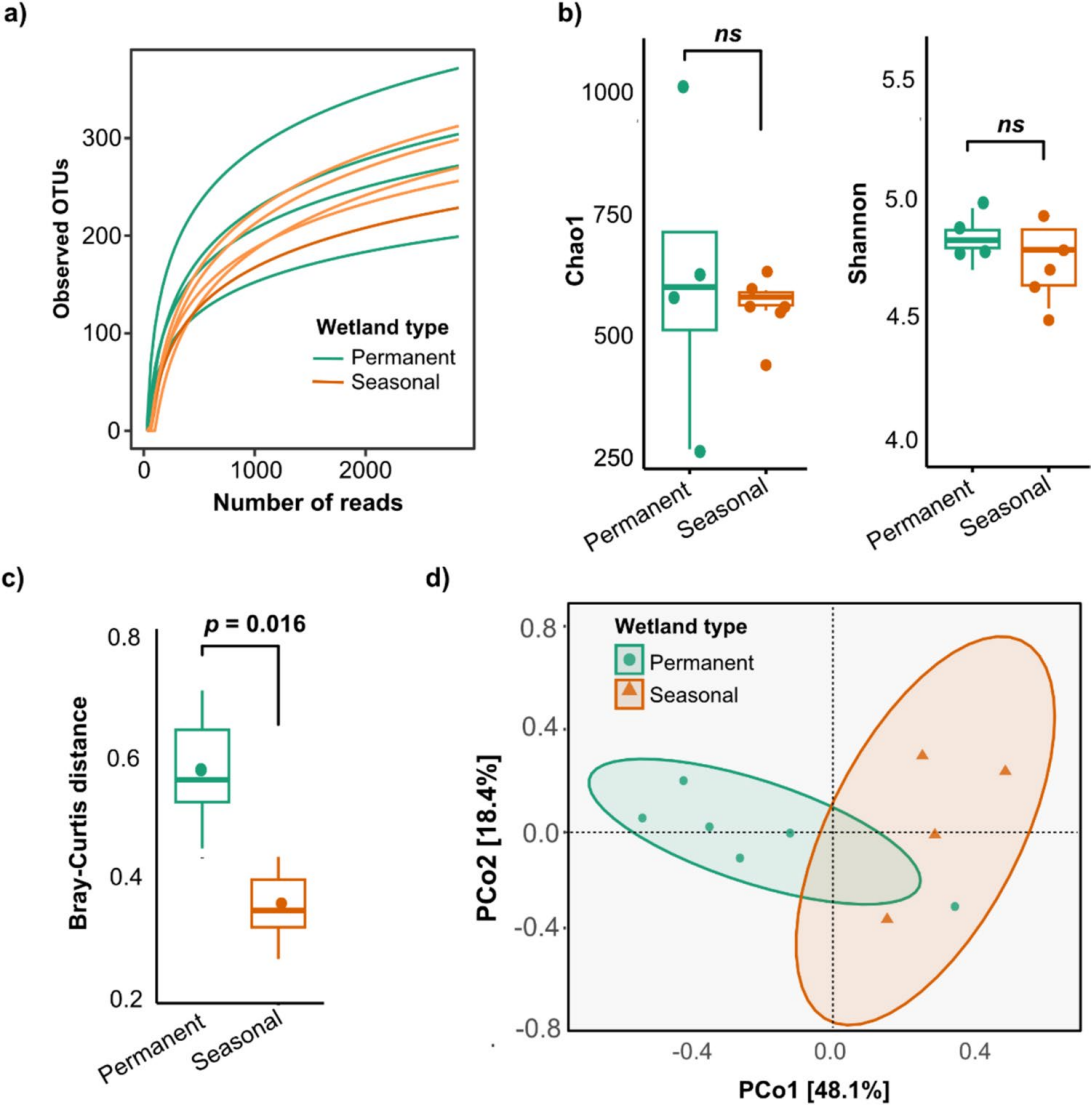
Diversity analysis of mycobiome composition in seasonal and permanent arid wetlands. **a** Rarefaction curves based on observed fungal OTUs identified in sediment samples from seasonal and permanent arid wetlands. **b** Boxplots comparing fungal alpha diversity indices (Chao1 and Shannon) between the two types of arid wetlands. **c** Boxplot showing significant differences in mycobiome beta diversity between seasonal and permanent arid wetland sediments. **d** Principal coordinate analysis (PCoA) based on Bray–Curtis distances. The significance of the separation of samples in ordination space was confirmed by the adonis PERMANOVA test (*F* = 3.733, *R*^*2*^ = 0.3181, *p* = 0.0031) and ANOSIM (*R* = 0.4802, *p* = 0.0230)

Taxonomic classification of the assembled contigs revealed a strong bacterial dominance (93.3% to 98.8% of classified reads), while eukaryotic reads—primarily fungi and protists—comprised 0.99%–6.67%, with viral reads being the least abundant (0.08% to 0.46%). This is consistent with several studies that have reported higher abundance bacterial taxa in similar environments (Wu et al. 2024; Tidimalo et al. 2024). A total of 409 fungal OTUs were identified from 201,304 fungal sequences, spanning eight phyla, 34 classes, 63 orders, 84 families, and 132 genera (Supplementary Material Table S2). Rarefaction curves of the observed OTUs exhibited asymptotic trends (Fig. 1a), indicating sufficient sequencing depth capturing the fungal diversity present in both wetland types (Cameron et al. 2021).

### Compositionally distinct mycobiomes despite similar alpha diversity across wetland types

Alpha diversity metrics, Chao1 (richness) and Shannon index (diversity), did not significantly differ between wetland types (Wilcoxon rank-sum test, *p* > 0.05) (Fig. 1b). In contrast, beta diversity analysis based on Bray–Curtis dissimilarity revealed distinct fungal community structures (Fig. 1c). PCoA further supported this separation, with PC1 and PC2 explaining 48.1%, and 18.4%) of the variation (Fig. 1d). Statistical tests confirmed significant compositional differences between wetland types (PERMANOVA: *F* = 3.733, *R*^*2*^ = 0.3181, *p* = 0.0031; ANOSIM: *R* = 0.4802, *p* = 0.023). These results suggest that while richness and diversity were similar, fungal communities were compositionally distinct, likely shaped by environmental filtering that favors different taxa under seasonal versus permanent wetland conditions.

Betadisper analysis (*F* = 0.0677, *p* = 0.81) indicated similar within-group dispersion, affirming that the observed compositional shifts stem from true ecological differentiation rather than stochastic variability among replicates. Notably, these findings align with observations by Park et al. (2020), who reported strong compositional variation in fungal communities among geographically distinct Korean wetlands, despite varying levels of alpha diversity. While our study found equivalent diversity across sites, both studies converge on the insight that distinct wetland environments structure unique fungal assemblages. This underscores the importance of assessing taxonomic composition in addition to diversity metrics, as ecologically or functionally divergent communities can exhibit similar diversity profiles (Omidipour et al. 2021).

### Hydrology-driven taxonomic divergence in arid wetland mycobiomes

**Fig. 2:**
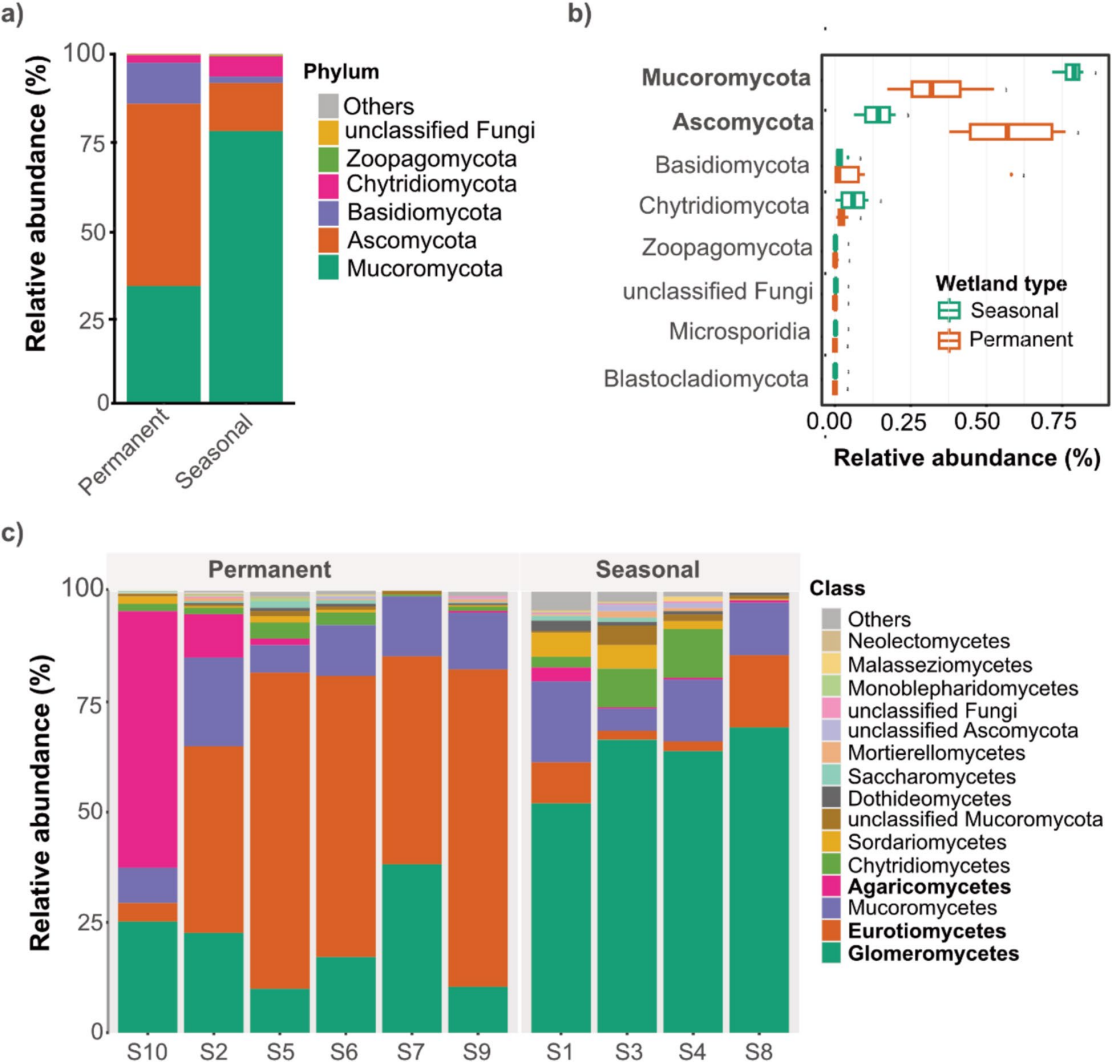
Mycobiome variation in the sediment from seasonal and permanent arid wetlands. **a** Stacked bar plot illustrating the relative abundance of the dominant fungal phyla detected. **b** Boxplots comparing the relative abundance of major phyla between seasonal and permanent arid wetlands. *Mucoromycota* (Wilcoxon rank-sum test, *p* = 0.007) and *Ascomycota* (Wilcoxon rank-sum test, *p* = 0.011), which exhibited significantly higher abundance in seasonal and permanent arid wetlands, respectively, are highlighted in bold. **c** Stacked bar plot showing the relative abundance of major fungal classes. Taxa having significantly higher abundance (Wilcoxon rank-sum test, *p* < 0.05) in seasonal and permanent arid wetlands are highlighted in bold

Fungal communities in the arid wetland sediments exhibited distinct taxonomic compositions, likely driven by hydrological regimes and associated environmental conditions (Gionchetta et al. 2019). At the phylum level, community composition diverged markedly between wetland types: *Mucoromycota* dominated seasonal wetlands (77.9%) but was considerably less abundant in permanent wetlands (33.5%), whereas *Ascomycota* exhibited the opposite trend (13.8% vs. 52.2%). *Basidiomycota* (2.3% vs. 11.7%) and *Chytridiomycota* (5.8% vs. 2.2%) were present in lower proportions, while minor phyla—*Zoopagomycota*, *Microsporidia*, and *Blastocladiomycota*—were detected at trace levels. A small fraction of sequences remained unclassified (0.3% in seasonal and 0.1% in permanent wetlands). Statistical comparisons (Wilcoxon rank-sum test, *p* < 0.05) confirmed significantly greater *Mucoromycota* abundance in seasonal wetlands and higher Ascomycota prevalence in permanent sites (Fig. 2a–b, Supplementary Fig. S1), a pattern consistent with findings from hydrologically variable wetland ecosystems (Chen et al. 2024).

**Fig. 3:**
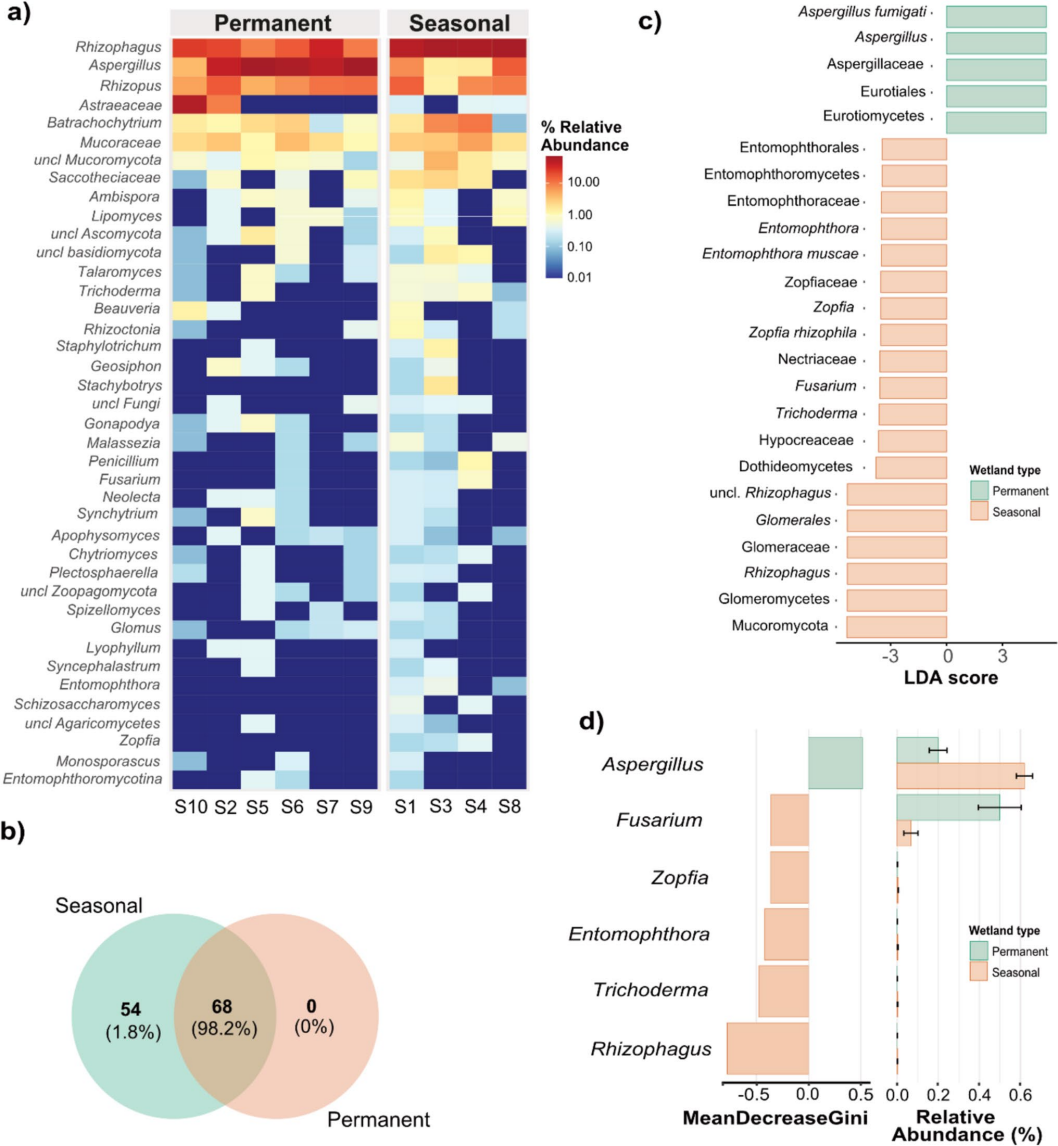
Genus-level mycobiome community composition in arid wetland sediments. **a** The genus-level distribution of the top 40 fungal OTUs. **b** A Venn diagram illustrates the shared and unique core fungal OTUs between seasonal and permanent arid wetland samples, providing insights into taxa exclusive to each habitat. **c** LefSe analysis of fungal biomarkers indicating statistically different (log_10_ LDA score > 3, *p* < 0.05) mycobiome groups in the seasonal and permanent arid wetland samples. **d** Random forest (RF) analysis ranking fungal genera by their importance in distinguishing between seasonal and permanent arid wetlands, based on their mean decrease in accuracy (MeanDecreaseGini)

This phylum-level shift provides a foundation for deeper resolution of taxonomic patterns. At the class level, eight dominant classes—*Glomeromycetes*, *Eurotiomycetes*, *Mucoromycetes*, *Agaricomycetes*, *Chytridiomycetes*, *Sordariomycetes*, unclassified *Mucoromycota*, and *Dothideomycetes*—together accounted for over 96% of total fungal sequences across both wetland types (Fig. 2c). Seasonal wetlands were significantly enriched in *Glomeromycetes* (arbuscular mycorrhizal fungi), *Chytridiomycetes*, *Sordariomycetes*, unclassified *Mucoromycota*, and *Dothideomycetes*, with *Glomeromycetes* showing the most marked enrichment (*p* < 0.001). In contrast, *Eurotiomycetes* (50.2%) and *Agaricomycetes* (11.7%) were more abundant in permanent wetlands (*p* < 0.01), highlighting class-level distinctions aligned with broader hydrological influences. Genus-level analysis further underscored these ecological divergences (Fig. 3). *Rhizophagus* (*Glomeromycetes*), *Rhizopus* (*Zygomycetes*), unclassified *Mucoraceae*, unclassified *Astraeaceae* (*Agaricomycetes*), and *Aspergillus* (*Eurotiomycetes*) emerged as dominant taxa, each exceeding 1% relative abundance and together accounting for over 89% of the total fungal community. Seasonal wetlands were characterized by elevated abundances of *Rhizophagus* (62%), *Rhizopus* (8.6%), unclassified *Mucoraceae* (3.5%), and *Batrachochytrium* (5.1%), suggesting adaptation to dynamic and resource-variable conditions. Conversely, permanent wetlands were strongly dominated by *Aspergillus* (50%) and unclassified *Astraeaceae* (11.2%), with *Rhizophagus* (20%) and *Batrachochytrium* (1.4%) also present at moderate levels.

These taxonomic distinctions reflect fundamental ecological responses of fungal communities to contrasting wetland hydrological regimes. This hydrology-mediated divergence at multiple taxonomic levels underscores the role of water availability as a key ecological filter shaping fungal community composition in arid wetland sediments. This is consistent of findings of the several studies in wetland ecosystems worldwide (Gionchetta et al. 2019; Brooks et al. 2023; Wu et al. 2024) It also highlights that shifts in wetland hydrology, whether due to climate change or anthropogenic alteration, could restructure mycobiome composition and, by extension, impact essential ecosystem functions such as organic matter turnover, nutrient cycling, and plant-fungal symbioses.

### Core mycobiome and habitat-specific indicators reflect functional adaptation to hydrological regime

A core microbiome analysis (Fig. 3b) identified 68 OTUs, comprising 98.2% of total fungal sequences, shared between seasonal and permanent wetlands. This shared core likely consists of generalist fungi adapted to the overarching arid wetland conditions (Sriswasdi et al. 2017). In contrast, 54 unique OTUs (1.8%) were exclusive to seasonal wetlands, suggesting the presence of specialist taxa adapted to the episodic hydrological disturbances characteristic of these environments (Arias-Real et al. 2023; Chen et al. 2023; Graça et al. 2024). LefSe and Random Forest analyses further delineated habitat-specific indicators. *Rhizophagus*, *Trichoderma*, *Fusarium*, *Entomophthora*, and *Zopfia* were enriched in seasonal wetlands, while Aspergillus was the dominant indicator genus in permanent wetlands (Fig. 3c–d). These findings are consistent with the phylum-to-genus trends presented in “Hydrology-driven taxonomic divergence in arid wetland mycobiomes” section but offer deeper insight into ecological roles and adaptive strategies.

In seasonal wetlands *Rhizophagus* (AMF) in seasonal wetlands suggests a functional adaptation to periodic desiccation and nutrient pulses (Guillén et al. 2019), the symbiotrophic interactions supporting host plant resilience via enhanced nutrient and water uptake during transient wet phases (Li et al. 2019; Fresno et al. 2023). Co-occurring taxa such as *Trichoderma* (a mycoparasite and plant growth promoter), and *Fusarium* (with saprophytic, pathogenic, or endophytic lifestyles) point to complex ecological interactions shaped by fluctuating moisture regimes and variable biotic resources (Poveda 2021; Martínez-Arias et al. 2022). The detection of insect pathogen *Entomophthora muscae, Batrachochytrium* and the halotolerant *Zopfia rhizophila* suggests additional specialization to insect-, amphibian-host and osmotic stress conditions, respectively (Heard et al. 2014; Dwiastuti et al. 2024; Barnés-Guirado et al. 2025). In contrast, permanent wetlands favored taxa such as *Aspergillus* and unclassified *Astraeaceae* (*Agaricomycetes*), whose metabolic versatility and ligninolytic capacity, respectively, reflect adaptation to more stable, oxygen-variable, and organic-rich environments (Liers et al. 2011; Bueno de Mesquita et al. 2024). These taxa likely contribute to carbon turnover through the degradation of recalcitrant organic matter, which is more consistently available in these systems.

### Hydrological regime and solute dynamics drive fungal community differentiation

**Fig. 4:**
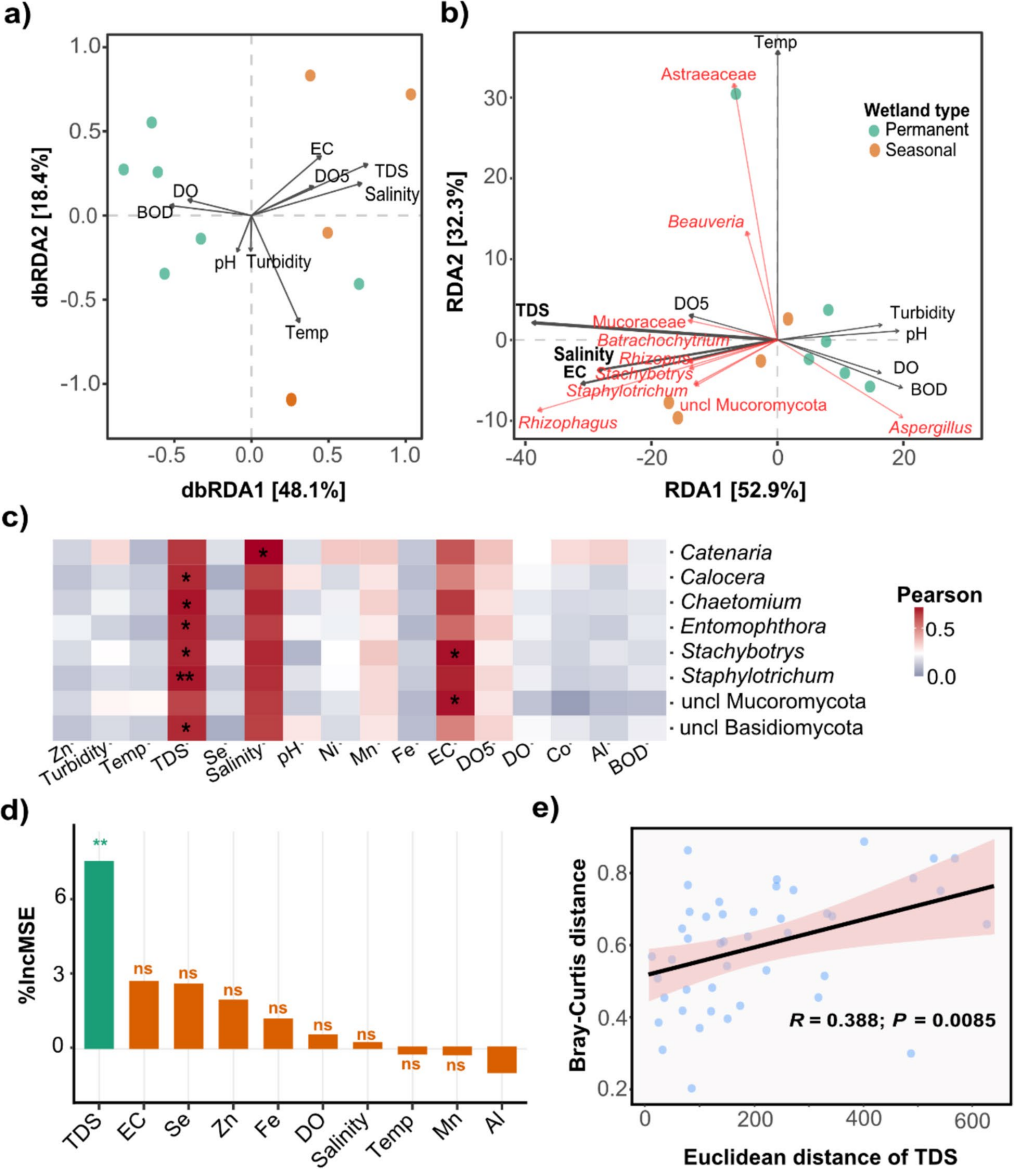
Environmental Drivers of Fungal Community Composition. **a** Distance-based redundancy analysis (dbRDA) of environmental variables in seasonal and permanent wetlands. Arrows represent environmental factors, with direction and proximity indicating correlation strength. **b** RDA plot showing relationships between environmental parameters and fungal community composition. **c** Pearson correlation analysis between fungal genera and environmental factors (**p < 0.01, * p < *0.05*). **d** Random forest modeling identifying TDS as the sole significant predictor of fungal composition based on percent increase in the mean squared error (%*IncMSE*) at *p* < *0.01*. e) Regression analysis of Euclidean distance of total dissolved solids (TDS) vs. Bray–Curtis distance, highlighting the impact of TDS on fungal community structure

To elucidate the environmental determinants shaping fungal beta diversity across arid wetland types, we employed distance-based redundancy analysis (dbRDA; Fig. 4a). Consistent with results of PCoA (Fig. 2a), dbRDA revealed clear separation of samples (PERMANOVA, *F* = 2.481, *p* = 0.009; ANOSIM, *R* = 0.293, *p* = 0.037). The primary ordination axis (dbRDA1) explained 48.1% of the variation in fungal composition (*R*^*2*^ = 0.4801, *p* < 0.01), identifying wetland type—and by extension, hydrological regime—as the dominant structuring factor. The secondary axis (dbRDA2), explaining an additional 18.4%, captured the influence of physicochemical parameters linked to these regimes.

Permanent wetlands showed elevated levels of dissolved oxygen (DO), biological oxygen demand (BOD5), and pH (Fig. 4b), indicative of oxygen-rich conditions with active organic matter turnover (Yan et al. 2024; Chen et al. 2024). Seasonal wetlands, in contrast, exhibited higher electrical conductivity (EC), total dissolved solids (TDS), and salinity, characteristic of fluctuating, solute-rich environments (Austin et al. 2004; Chen et al. 2024). These differences reflect dynamic dry–wet cycles typical of ephemeral wetlands. Statistical modeling identified TDS, EC, and salinity as the most influential predictors of beta diversity (PERMANOVA *R*^*2*^ = 0.49, 0.32, and 0.29, respectively; *p* < 0.01), underscoring the role of ionic composition in shaping fungal communities.

Collectively, these patterns highlight the role of hydrological regime as a primary environmental filter, shaping fungal community composition through its effects on oxygen levels, salinity, moisture availability, and resource dynamics (Kraft et al. 2015; Peipoch et al. 2019). The coexistence of a shared core and distinct habitat-specific taxa indicates a dual strategy of generalist persistence and specialist adaptation, underpinned by niche partitioning and functional redundancy. These findings underscore the ecological resilience and functional complexity of mycobiomes in arid wetland systems.

Taxon-specific responses further delineated the ecological differentiation (Fig. 4b). In permanent arid wetlands, *Aspergillus* positively correlated with BOD, DO, pH, and turbidity. While BOD and DO are often inversely related, their concurrent association with *Aspergillus* may reflect its metabolic flexibility and ability to thrive during early stages of organic matter decomposition, when both oxygen availability and microbial respiration are high (Onufrak et al. 2020; Yan et al. 2024). This pattern indicates a preference for moderately disturbed, oxygen-rich environments with active carbon cycling. In contrast, fungal genera such as *Rhizophagus*, *Batrachochytrium*, *Stachybotrys*, *Staphylotrichum*, and *Rhizopus*, along with unclassified *Mucoromycota*, demonstrated a strong positive associations with salinity and EC, suggesting physiological adaptation to the osmotic stress prevalent in seasonal wetlands (Wu et al. 2013; Chen et al. 2023). These contrasting patterns underscores findings suggests environmental filtering of fungal taxa based on salinity tolerance and organic matter decomposition potential, consistent with ecological theories of microbial adaptation to environmental gradients in wetland ecosystems (Wu et al. 2013; Peipoch et al. 2019; Wan et al. 2021).

Mantel tests further elucidated taxon–environment relationships by evaluating correlations between fungal genera and individual environmental variables (Fig. 4c). While dominant taxa such as *Rhizophagus, Rhizopus, Aspergillus,* and *Batrachochytrium* did not exhibit statistically significant correlations (− 0.6 < *ρ* > 0.6, *p* > 0.05), several less dominant but ecologically relevant taxa displayed significant associations. In particular, positive correlations were observed between TDS and genera including *Calocera*, *Chaetomium,* Entomophthora, *Stachybotrys, Staphylotrichum,* and unclassified *Mucoromycota* (*p* = 0.035, 0.018, 0.042, 0.015, 0.009, and 0.021, respectively). Additionally, *Catenaria* was positively associated with EC, while both *Stachybotrys* and unclassified *Mucoromycota* correlated significantly with elevated levels of both EC and TDS. These findings reinforce the hypothesis that ionic strength and osmotic stress, reflected in EC and TDS values, are critical environmental determinants of fungal community structure in arid wetlands. Supporting this, random forest modeling identified TDS as the sole significant predictor of fungal community composition (*p* < 0.01; Fig. 4d). Complementary regression analysis of Euclidean distance versus Bray–Curtis dissimilarity further underscored the influence of TDS, revealing a statistically significant relationship (*R*^*2*^ = 0.388, *p* = 0.0085; Fig. 4e).

In summary, our findings demonstrate a clear ecological link between hydrological regime and fungal community structure in arid wetland systems. Hydrological variability acts as a strong environmental filter by modulating key physicochemical parameters, particularly salinity, ionic composition, and oxygen availability which in turn drive fungal community assembly (Kraft et al. 2015). Fungal taxa exhibit pronounced niche partitioning along these hydrochemical gradients. For instance, *Aspergillus* is favored in relatively stable, oxygen-rich conditions, whereas *Rhizophagus* and members of *Mucoromycota* are better adapted to environments characterized by high salinity and osmotic stress, typical of seasonal wetlands (Crowther et al. 2014; Mueller et al. 2016). The consistent identification of TDS as a primary structuring factor underscores the importance of solute dynamics in shaping microbial ecology within hydrologically variable landscapes. These insights are crucial for understanding fungal responses to both natural fluctuations and anthropogenic pressures, such as water abstraction and climate-induced drying, which may significantly alter wetland salinity regimes.

### Functional analysis of fungal community in the arid wetlands

**Fig. 5:**
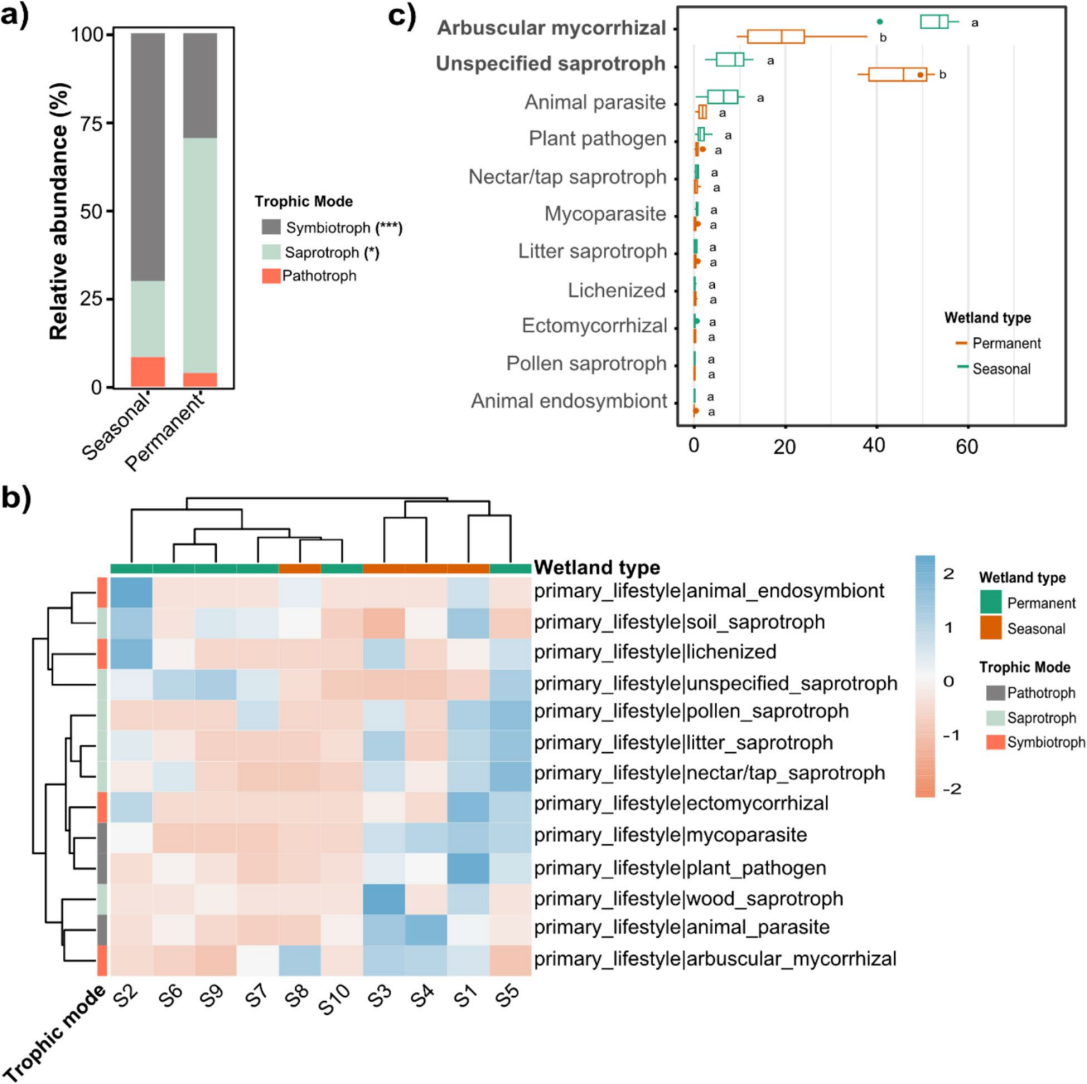
Functional prediction and annotation of fungal communities in permanent and seasonal arid wetland sediments using FUNGuild. **a** Stacked bar plot depicting the relative abundance of fungal taxa across three trophic modes. Trophic modes with significant differences in relative abundance are indicated in brackets (Wilcoxon rank-sum test: *** *p* < 0.001, ** *p* < 0.01, * *p* < 0.05). **b** Clustered heatmap illustrating functional predictions and annotations for the 13 predominant fungal guilds. **c** Box plot representing the relative abundance of fungi across 11 primary functional guilds. Guilds exhibiting significant differences in relative abundance between permanent and seasonal arid wetlands are highlighted in bold (Wilcoxon rank-sum test, *p* < 0.05)

To elucidate the functional composition of fungal communities in these arid wetlands, we employed FUNGuild (Nguyen et al. 2016) for functional annotation, categorizing fungal taxa into symbiotrophs, saprotrophs, and pathotrophs, further subdivided into 67 distinct guilds (Supplementary Material Table S3). This analysis revealed significant differences in functional structure between permanent and seasonal arid wetlands (Fig. 5), mirroring the taxonomic differences observed previously.

Statistical analyses demonstrated a pronounced divergence in fungal functional strategies. Permanent wetlands exhibited a significantly higher abundance of saprotrophs (59.7% vs. 21.5%; Wilcoxon rank-sum test, *p* < 0.05) (Fig. 5a), reflecting a greater reliance on organic matter decomposition under stable moisture conditions. This functional enrichment aligns with the higher abundance of Ascomycota, particularly *Aspergillus* (Fig. 3), in these wetlands, consistent with previous studies identifying Ascomycota as dominant in similar ecosystems (Onufrak et al. 2020; Xie et al. 2020; Yan et al. 2024). Conversely, seasonal wetlands were characterized by an enrichment of symbiotrophs (69.3% vs. 36.1%; Wilcoxon rank-sum test, *p* < 0.001), particularly AMF (e.g., *Rhizophagus*), suggesting an ecological strategy that enhances nutrient acquisition and stress tolerance under fluctuating hydrochemical conditions (Guillén et al. 2019; Chen et al. 2024). This dominance of AMF in seasonal wetlands reinforces the importance of symbiotic interactions for plant resilience in ephemeral wetlands, where AMF colonization increases in response to hydrological variability (Guillén et al. 2019; Chen et al. 2024). While pathotrophic fungi (animal parasites, plant pathogens, and mycoparasites) were more abundant in seasonal wetlands (9.2% vs. 4.3%), this difference was not statistically significant (*p* = 0.181). However, the presence of these pathotrophs (e.g., *Mortierella*, *Cryptococcus*, *Exophiala*, *Candida*, *Microsporidium*, *Fusarium*, *Penicillium*) in both environments suggests a potential role in disease dynamics and trophic interactions within these wetland ecosystems (Fig. 5b,c). Other guilds, like animal parasites (6.9% vs. 3.42%) and soil saprotrophs (10.8% vs. 11.5%), showed minor, non-significant variations between wetland types. Notably, unspecified saprotrophs were significantly more abundant in permanent wetlands (46.8% vs. 8.7%; Wilcoxon rank-sum test, *p* < 0.01).

These functional differences are clearly linked to the distinct hydrological regimes and associated environmental conditions in each wetland type. The stable moisture conditions in permanent wetlands favor saprotrophic fungi, which play a crucial role in nutrient cycling through decomposition. The fluctuating water availability in seasonal wetlands, on the other hand, selects for symbiotrophic fungi, particularly AMF, which enhance plant access to nutrients and water under these stressful conditions (Read 2003). This functional divergence reflects niche partitioning, where different fungal groups specialize in different ecological roles based on the prevailing environmental conditions. The enrichment of *Aspergillus* in permanent wetlands, coupled with its known metabolic versatility (Andersen et al. 2008; Paulussen et al. 2017), further underscores the importance of niche partitioning based on resource availability and other environmental factors. The presence of pathotrophs in both wetland types, while not statistically different in abundance, suggests that these fungi may play important roles in regulating populations and energy flow within the food web, although further investigation is needed to determine the extent of their impact.

In summary, our findings highlight the significant influence of wetland type, driven by distinct hydrological regimes, on both fungal community composition and function. Seasonal wetlands support a higher diversity of symbiotrophic strategies, particularly AMF, reflecting adaptations to fluctuating hydrochemical conditions and the importance of plant-fungal symbioses. Permanent wetlands, with their more stable moisture regimes, are dominated by saprotrophic fungi, emphasizing the importance of decomposition and nutrient cycling in these systems. These patterns underscore the role of hydrological regimes in shaping fungal assemblages and suggest that fungal taxa exhibit functional specialization in response to the environmental gradient characteristic of these arid wetland ecosystems. However, it is important to acknowledge that the limited number of wetland samples (n = 10) may constrain the generalizability of our conclusions. While the observed patterns provided valuable preliminary insights into fungal ecological strategies in arid wetlands, future research should incorporate larger and more temporally distributed datasets to enhance statistical robustness and capture intra-seasonal variability. Furthermore, elucidating the mechanistic pathways through which these functionally diverse fungal communities contribute to biogeochemical cycling and ecosystem resilience under climate-driven hydrological change remains a critical priority for advancing our understanding of these vulnerable ecosystems. Understanding these linkages is crucial for predicting how these vital ecosystems will respond to future environmental change.

## Conclusion

This study used shotgun metagenomics to investigate fungal diversity, functional guilds, and environmental drivers in arid seasonal and permanent wetlands of South Africa. Fungal communities exhibited distinct taxonomic and functional profiles between wetland types, primarily shaped by hydrological regime and associated hydrochemical gradients, especially total dissolved solids (TDS) and salinity. Seasonal wetlands, subject to fluctuating moisture conditions, were enriched in symbiotrophs such as *Rhizophagus* and *Zopfia rhizophila*, reflecting adaptations to osmotic stress and drought cycles. In contrast, permanent wetlands supported higher abundances of saprotrophic taxa like *Aspergillus fumigatus*, consistent with more stable, decomposition-friendly conditions. While alpha diversity remained similar across sites, beta diversity metrics confirmed significant structural divergence between wetland types. RF modeling and Mantel tests highlighted TDS and salinity as key predictors of community composition, with specific taxa showing strong environmental associations. Functional analysis revealed a clear bifurcation: symbiotrophs dominated in seasonal systems, while saprotrophs were more abundant in permanent ones. Pathotrophs occurred in both but were less influential overall. These results emphasize the role of environmental filtering and niche differentiation in structuring fungal communities under extreme conditions. The identification of indicator taxa such as *Rhizophagus* and *Stachybotrys* offers a basis for monitoring ecosystem responses to hydrological change. Importantly, maintaining or mimicking natural water regimes may enhance fungal functional diversity and resilience. This study contributes to understanding microbial adaptation in arid wetlands and underscores the need to integrate fungal dynamics into conservation and restoration strategies under increasing climate pressure.

## Supplementary Information

Below is the link to the electronic supplementary material.

Supplementary file1 (XLSX 46 KB)

ESM 2 (PNG 1.09 MB)

High Resolution Image (TIF 1.86 MB)

## Data Availability

Raw fastq datasets associated with this study has been deposited at the NCBI database under BioProject ID PRJNA972844, with SRA accession numbers SRX20358958–SRX20358949. All other data generated are included within the article and supplementary materials.
