# Supplementary figures and images for: Hydrological regime and niche partitioning drive fungal community structure and function in arid wetlands sediments of South Africa
Source: Environ Sci Pollut Res Int. 2025 Jun 6;32(25):15217–31. doi: 10.1007/s11356-025-36592-0 (PMC12202646; doi:10.1007/s11356-025-36592-0)

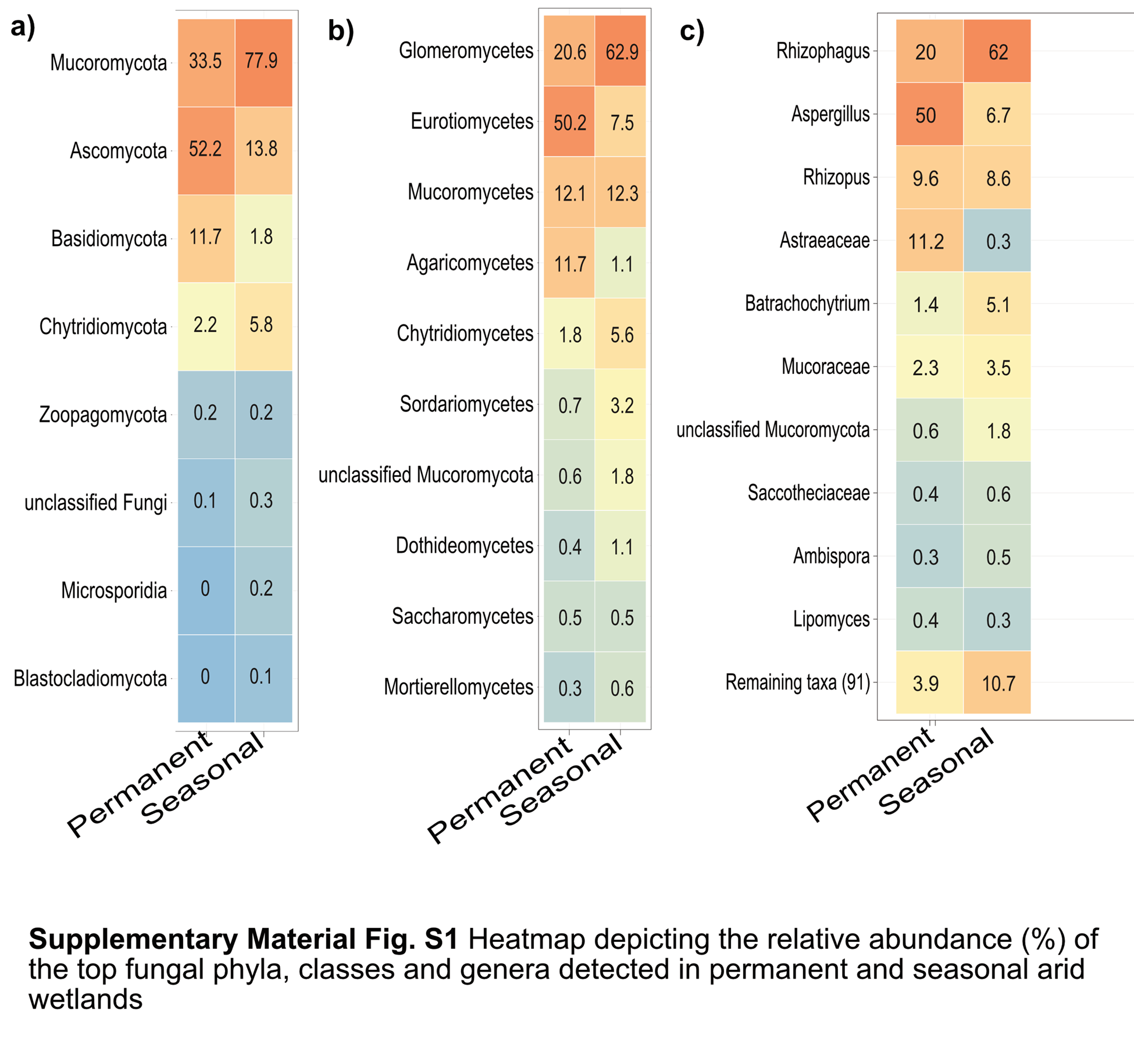

Supplement: Supplementary file 2 — (PNG 1.09 MB) [file 11356_2025_36592_Fig6_ESM.png]

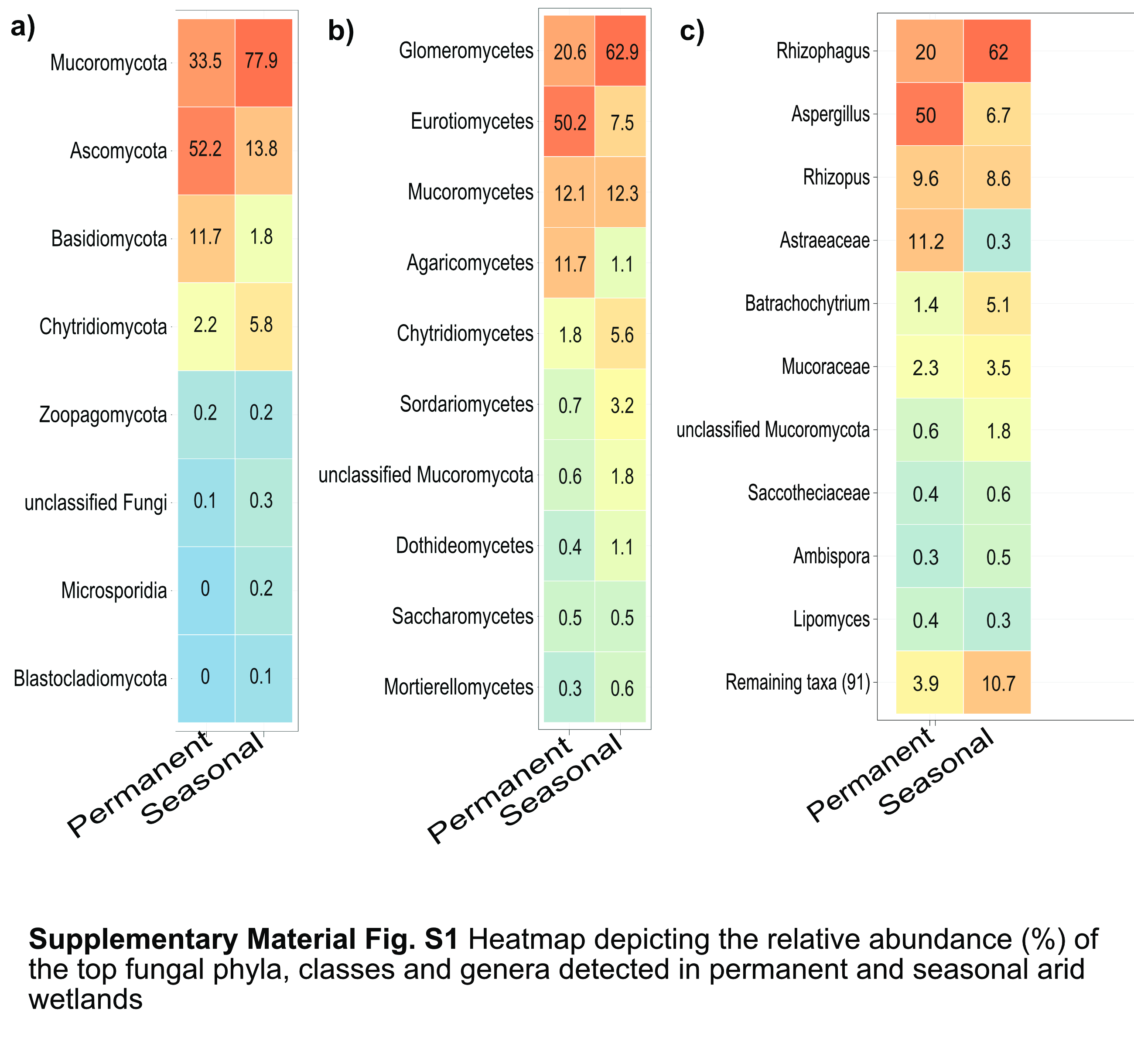

Supplement: Supplementary file 3 — High Resolution Image (TIF 1.86 MB) [file 11356_2025_36592_MOESM2_ESM.tiff]
